# Personal—Our Colleague

**Published:** 1884-07

**Authors:** 


### PERSONAL—OUR COLLEAGUE.

Dr. W. D. Miller landed at New York from Bremen, Friday P. M., June 20th. Due preparation had been made to welcome him, and on Saturday evening he was tendered a complimentary dinner at the Union League Club, by a few gentlemen who are familiar with the special work in which he has been engaged. Dr. Miller's stay was too brief to allow time for much consultation, and the necessities of the case limited the number taking part in it to a very few. This was a source of regret, but was unavoidable. One of those present, a former classmate of the guest of the evening, sends us a brief notice of the dinner, which will be found among the items of "Current News."

He was the guest of the dentists of Buffalo on Monday evening, and on Tuesday evening was entertained at dinner by members of the Microscopical Society of the same place. He sails for home on the 23d inst., and in the meantime has friends and relatives to visit whom he has not seen for years.

We speak of these personal matters, not in a sycophantic spirit, but because many friends and admirers of Dr. Miller have expressed a desire to meet him, while the limit of his time in this country utterly precludes the possibility of his making visits to professional brethren who do not live on the line of his necessary travel between New York and his native place—Newark, Ohio.

